# Effects of esketamine combined with QLB in older frail patients with colorectal cancer surgery

**DOI:** 10.3389/fmed.2026.1749820

**Published:** 2026-03-17

**Authors:** Shuhong Dai, Liwei Wang, Keshuai Zhang, Rongguo Wang, Na Li, Xu Zhang

**Affiliations:** 1 Department of Anesthesiology, Xuzhou Central Hospital, Xuzhou, China; 2 Department of Anesthesiology, Xuzhou Children’s Hospital, Xuzhou, China

**Keywords:** cognitive function, colorectal cancer surgery, depression, elderly frail patients, esketamine, postoperative anxiety, quadratus lumborum block

## Abstract

**Objective:**

To investigate the effects of esketamine combined with quadratus lumborum block (QLB) on postoperative anxiety, depression, and cognitive function in frail older patients undergoing radical colorectal cancer surgery.

**Methods:**

This study included older frail patients scheduled for radical colorectal cancer surgery. Patients were randomly assigned to either the control group, receiving standard anesthesia, or the combination group, receiving QLB combined with esketamine anesthesia. Hemodynamic parameters, anesthetic drug dosages, recovery quality, cognitive function, and pain levels were compared between the two groups.

**Results:**

Both groups showed no significant differences in mean arterial pressure (MAP) and heart rate (HR) before anesthesia induction (*p* > 0.05). However, after induction, during pneumoperitoneum, and postoperatively, the combination group exhibited significantly lower MAP and HR compared to the control group (*p* < 0.05). The combination group required lower dosages of remifentanil and propofol (*p* < 0.05), had shorter recovery times and lower agitation scores upon awakening (*p* < 0.05). Additionally, the combination group had significantly higher Mini-Mental State Examination (MMSE) scores on postoperative days 1 and 3, and lower Visual Analog Scale (VAS) scores for pain at 1, 4, and 12 h post-extubation (*p* < 0.05).

**Conclusion:**

Esketamine combined with QLB in Olderfrail patients undergoing radical colorectal cancer surgery stabilizes hemodynamic parameters, reduces anesthetic drug usage, improves recovery quality, mitigates cognitive dysfunction, and alleviates pain. This combined approach is clinically beneficial and warrants further promotion.

## Introduction

Colorectal cancer is a prevalent malignancy worldwide, particularly affecting the older population (1, 2). Radical colorectal cancer surgery remains the cornerstone of treatment, offering the best chance for long-term survival (3, 4). However, older patients often present with frailty (5), characterized by decreased physiological reserves and increased vulnerability to stressors, which complicates their perioperative management (6, 7). Frailty in these patients is associated with higher rates of postoperative complications (7, 8), including anxiety, depression, and cognitive dysfunction (8, 9), all of which significantly impact their recovery and quality of life (10).

Anesthetic management plays a crucial role in mitigating these postoperative complications (11). Traditional anesthesia techniques, while effective in providing the necessary conditions for surgery, may not adequately address the specific needs of older frail patients (12). Recent advances in regional anesthesia, such as the quadratus lumborum block (QLB), have shown promise in enhancing postoperative recovery by providing superior analgesia and reducing the reliance on systemic opioids (13, 14), which are known to contribute to cognitive decline and mood disturbances (15, 16).

Esketamine, an enantiomer of ketamine, has gained attention for its unique properties, including rapid-acting antidepressant effects and minimal respiratory depression (17). Esketamine is increasingly used in surgeries associated with high stress and chronic pain risk, such as thoracic, orthopedic, and major abdominal surgeries (18). When used in combination with QLB, esketamine may offer a synergistic benefit, improving both the analgesic and psychological outcomes in the perioperative period (19). This combination could potentially stabilize hemodynamic parameters, reduce the required doses of other anesthetics, and enhance overall recovery quality.

Previous studies have demonstrated the benefits of QLB and esketamine individually in various surgical settings (19). However, there is a paucity of research examining their combined effects in older frail patients undergoing radical colorectal cancer surgery.

This study aims to fill this gap by investigating the impact of esketamine combined with QLB on postoperative anxiety, depression, and cognitive function in this vulnerable patient population. By addressing these aspects, our research seeks to contribute to the optimization of perioperative care for older frail patients, ultimately improving their surgical outcomes and quality of life.

## Methods

### Study design

This was a prospective, randomized, controlled trial conducted at Xuzhou Central Hospital. The study was approved by the institutional review board and all participants provided written informed consent.

### Participants

Older frail patients scheduled for radical colorectal cancer surgery between March 2023 and March 2024 were considered for inclusion. Frailty was assessed using the Fried Frailty Criteria (20), and eligible patients were those aged 65 years or older, with a frailty score of 3 or more. Inclusion Criteria: (1) Age ≥ 65 years; (2) Scheduled for radical colorectal cancer surgery; (3) Frailty score ≥ 3; (4) Provided informed consent. Exclusion Criteria: (1) Severe cardiovascular, hepatic, or renal dysfunction; (2) History of psychiatric illness or cognitive impairment unrelated to surgery; (3) Allergies to study medications; (4) Inability to provide informed consent; (5) Patients with American Society of Anesthesiologists (ASA) physical status ≥4.

### Sample size calculation

The required sample size was determined based on the primary outcome of MMSE scores at 24 h postoperatively. Drawing from prior research focusing on the patients undergoing non-cardiac thoracic surgery (21), where the mean MMSE was 15.0 ± 2.2, we aimed to detect a clinically significant improvement of 1.5 points in the combination group. With a two-sided significance level of 0.05 and a power of 80%, a minimum of 34 patients per group was identified. To account for a potential dropout rate of up to 15%, the final recruitment was set at 40 patients per group (total *N* = 80).

### Randomization and blinding

Patients were randomly assigned to either the control group or the combination group using a computer-generated randomization list. Allocation was concealed using sealed opaque envelopes. Both patients and outcome assessors were blinded to group assignments. However, the anesthesiologist administering the intervention was not blinded.

### Anesthesia methods

Both groups of patients were instructed to fast for 8 h before surgery. Upon entering the operating room, standard protocols were followed to establish intravenous access, administer low-flow oxygen via a face mask, and continuously monitor electrocardiogram (ECG), blood pressure, and oxygen saturation. The same anesthesiologist performed all anesthesia procedures to ensure consistency.

#### Anesthesia induction and airway management

All patients underwent standardized general anesthesia and monitoring. Induction was achieved with midazolam (0.1 mg/kg), fentanyl (2.5 ug/kg), and propofol (1.5–2.0 mg/kg). Cisatracurium besylate (0.15 mg/kg) was administered to facilitate endotracheal intubation. Following induction, all patients received an ultrasound-guided posterior QLB (QLB2). With the patient in the lateral decubitus position, a 2–5 MHz curved array transducer (SonoSite Edge II) was used to identify the quadratus lumborum (QL) muscle. A 21-gauge, 100-mm echogenic needle was advanced in-plane into the fascial plane between the QL muscle and the middle layer of the thoracolumbar fascia. Following negative aspiration, 0.375% ropivacaine (0.3 mL/kg) was injected.

#### Anesthesia maintenance and study interventions

Anesthesia was maintained using a continuous infusion of propofol (4–10 mg/kg/h) and remifentanil (0.1–0.3 ug/kg/min), titrated to maintain a Bispectral Index (BIS) between 40 and 60. Muscle relaxation was maintained with intermittent boluses of cisatracurium as required by the surgical procedure. The patients were randomized into two groups for the study intervention:

Control Group: Received the standardized anesthesia maintenance and QLB procedure as described above, with the addition of an equal volume of normal saline as a placebo, administered in the same manner as the esketamine infusion.

Combination Group: In addition to the standardized regimen, esketamine was administered intravenously as a 0.5 mg/kg bolus over 2 min before surgical incision, followed by a continuous maintenance infusion of 0.125 mg/kg/h until the conclusion of the surgery.

Hemodynamic stability was maintained within ±20% of baseline values. If Mean Arterial Pressure (MAP) decreased significantly, norepinephrine (4ug) was administered. Atropine (0.5 mg) was given for bradycardia (Heart Rate < 50 bpm).

### Outcome measures

#### Primary outcomes

Cognitive function was assessed using the Mini-Mental State Examination (MMSE) (22) at baseline, 24 h, and 72 h after surgery.

#### Secondary outcomes

- Hemodynamic stability, was assessed by measuring mean arterial pressure (MAP) and heart rate (HR) at baseline, post induction, during pneumoperitoneum and at the end of surgery.
- Total doses of propofol and remifentanil used.
- Pain levels, were measured using the Visual Analog Scale (VAS) (23) at 1, 4, 12, 24, and 48 h post-extubation.
- Recovery quality, including time to extubation, time to return of spontaneous respiration, and agitation scores upon awakening.

### Statistical analysis

Data were analyzed using R version 4.3.3 (2024-02-29). Continuous variables were expressed as mean ± standard deviation (SD) and compared using the independent t-test or Mann–Whitney U test as appropriate. Categorical variables were expressed as frequencies and percentages and compared using the chi-square test or Fisher’s exact test. Repeated measures ANOVA was used to analyze changes over time within groups. To quantify the clinical magnitude of the observed differences beyond mere statistical significance, effect sizes were reported as partial eta-squared (η^2^p). According to Cohen’s criteria, η^2^p values of 0.01, 0.06, and 0.14 were interpreted as small, medium, and large effects, respectively. A *p*-value of <0.05 was considered statistically significant.

### Ethical considerations

The study was conducted in accordance with the Declaration of Helsinki. Ethical approval was obtained from the Biomedical Research Ethics Review Committee of Xuzhou Central Hospital. Informed consent was obtained from all participants after explaining the purpose, procedures, risks, and benefits of the study.

## Results

### Participant characteristics

**Figure 1:**
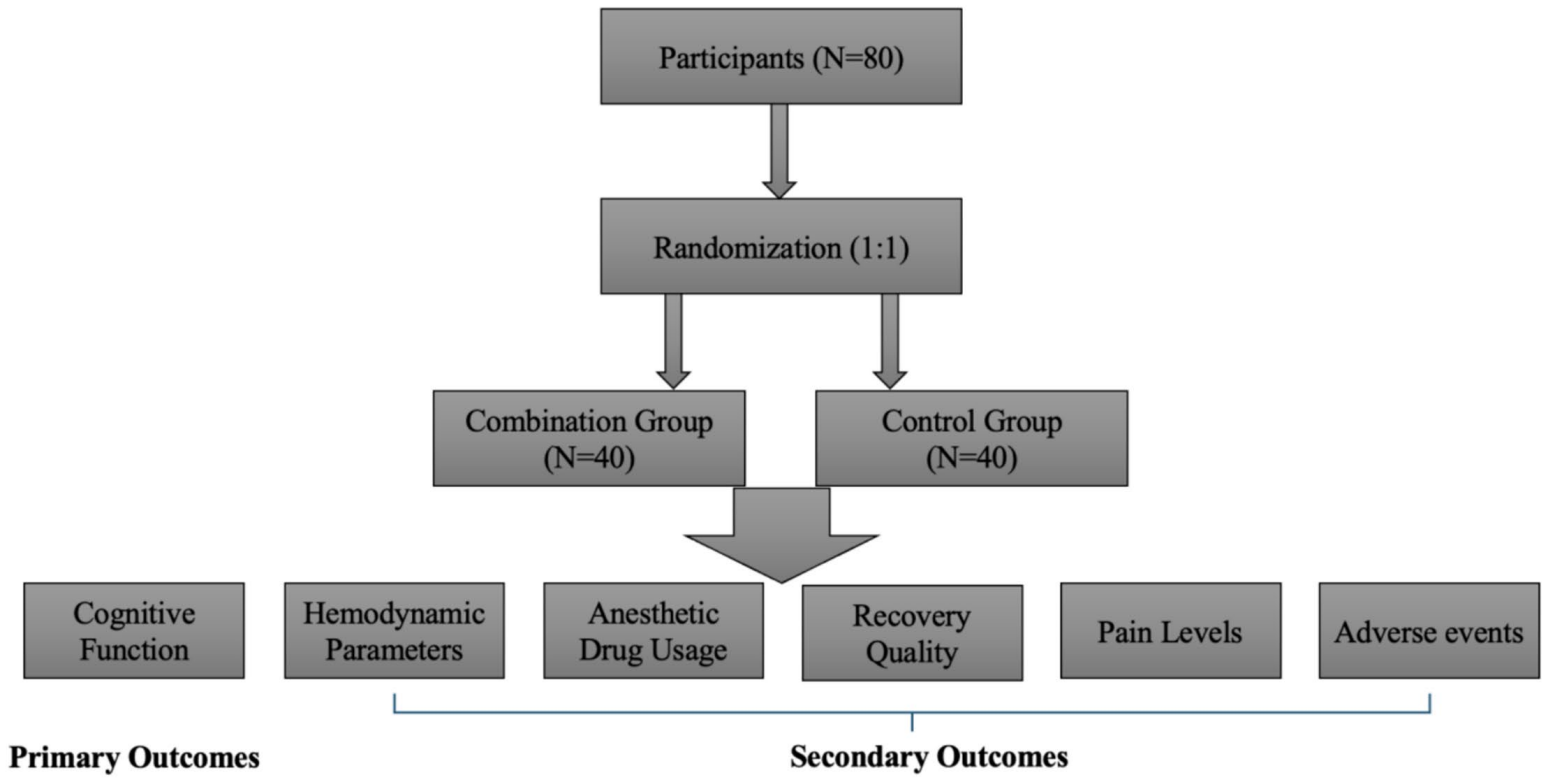
The whole design of the present study.

A total of 80 older frail patients were enrolled and randomly assigned to either the control group (*n* = 40) or the combination group (*n* = 40; Figure 1). Both groups were comparable in terms of age, gender distribution, body mass index (BMI), frailty scores, baseline MMSE scores, and ASA physical status. The two groups had no significant differences in baseline demographic and clinical characteristics (all *p* > 0.05; Table 1).

**Table 1:** Baseline characteristics of study participants.

Characteristic	Control group (*n* = 40)	Combination group (*n* = 40)	t/X^2^ value	*p-*value
Age (years)	72.5 ± 5.3	71.8 ± 6.1	0.548	0.585
Gender (M/F)	22/18	20/20	0.050	0.823
BMI (kg/m^2^)	23.1 ± 2.4	22.9 ± 2.7	0.350	0.727
Frailty score	3.6 ± 0.7	3.5 ± 0.8	0.595	0.554
Baseline MMSE score	27.2 ± 1.9	27.4 ± 2.1	−0.447	0.656
ASA physical status			0.087	0.957
1, n (%)	25 (62.5)	24 (60.0)		
2, n (%)	8 (20.0)	8 (20.0)		
3, n (%)	7 (17.5)	8 (20.0)		

M, male; F, female; BMI, body mass index; MMSE, Mini-Mental State Examination; ASA, American Society of Anesthesiologists.

### Hemodynamic parameters

Both groups showed stable hemodynamic parameters throughout the perioperative period. However, the combination group exhibited significantly lower MAP and HR during critical intraoperative periods (post-induction, during pneumoperitoneum, and postoperatively) compared to the control group (*p* < 0.05). Detailed hemodynamic data are presented in Table 2.

**Table 2:** Hemodynamic parameters in two groups.

Time point	Control group (*n* = 40)	Combination group (*n* = 40)	*t-*value	*p-*value	*η^2^p*
Baseline MAP (mmHg)	92.3 ± 8.1	91.7 ± 7.9	0.335	0.738	0.002
Post-induction MAP (mmHg)	85.4 ± 7.2	80.5 ± 6.8	3.129	0.002	0.231
During pneumoperitoneum MAP	88.7 ± 7.4	83.1 ± 6.9	3.501	0.001	0.343
Postoperatively MAP (mmHg)	90.2 ± 7.8	84.7 ± 7.2	3.277	0.002	0.245
Baseline HR (beats/min)	78.6 ± 6.3	77.9 ± 6.5	0.489	0.626	0.005
Post-induction HR (beats/min)	74.1 ± 5.7	70.3 ± 5.2	3.115	0.003	0.182
During pneumoperitoneum HR	75.6 ± 5.9	71.4 ± 5.5	3.293	0.001	0.406
Postoperatively HR (beats/min)	76.3 ± 6.1	72.0 ± 5.7	3.257	0.002	0.253

η^2^p indicates partial eta-squared, representing the effect size; values of 0.01, 0.06, and 0.14 represent small, medium, and large effects, respectively.

### Anesthetic drug usage

The combination group required significantly lower doses of remifentanil and propofol compared to the control group (*p* < 0.05). The reduced drug usage is detailed in Table 3.

**Table 3:** Anesthetic drug usage in two groups.

Drug	Control group (*n* = 40)	Combination group (*n* = 40)	*t-*value	*p-*value	*η^2^p*
Remifentanil (mg)	1.6 ± 0.4	1.1 ± 0.3	6.325	<0.001	0.339
Propofol (mg)	1,200 ± 150	900 ± 130	9.559	<0.001	0.539

η^2^p indicates partial eta-squared, representing the effect size; values of 0.01, 0.06, and 0.14 represent small, medium, and large effects, respectively.

### Recovery quality

Patients in the combination group had shorter extubation times and quicker return of spontaneous respiration compared to the control group. Agitation scores upon awakening were also significantly lower in the combination group (*p* < 0.05). These results are summarized in Table 4.

**Table 4:** Recovery quality in two groups.

Parameter	Control group (*n* = 40)	Combination group (*n* = 40)	*t-*value	*p-*value	*η^2^p*
Time to extubation (min)	18.5 ± 3.2	12.7 ± 2.9	8.494	<0.001	0.352
Time to spontaneous respiration (min)	20.4 ± 3.7	14.1 ± 3.1	8.255	<0.001	0.527
Agitation score upon awakening	4.2 ± 0.6	2.8 ± 0.5	11.337	<0.001	0.363

η^2^p indicates partial eta-squared, representing the effect size; values of 0.01, 0.06, and 0.14 represent small, medium, and large effects, respectively.

### Cognitive function

MMSE scores were significantly higher in the combination group on postoperative days 1 and 3 compared to the control group, indicating better preservation of cognitive function (*p* < 0.05). Detailed MMSE scores are shown in Table 5.

**Table 5:** Cognitive function (MMSE scores) in two groups.

Time point	Control group (*n* = 40)	Combination group (*n* = 40)	*t-*value	*p-*value	*η^2^p*
Baseline	27.2 ± 1.9	27.4 ± 2.1	−0.447	0.656	0.003
Postoperative Day 1	25.1 ± 2.0	27.0 ± 2.2	−4.042	<0.001	0.173
Postoperative Day 3	26.0 ± 1.8	28.1 ± 2.0	−4.936	<0.001	0.238

η^2^p indicates partial eta-squared, representing the effect size; values of 0.01, 0.06, and 0.14 represent small, medium, and large effects, respectively.

### Pain levels

VAS scores for pain were significantly lower in the combination group at all postoperative time points (1, 4, 12, 24, and 48 h) compared to the control group (*p* < 0.05), indicating better pain management. These results are presented in Table 6.

**Table 6:** Pain levels (VAS scores) in two groups.

Time point	Control group (*n* = 40)	Combination group (*n* = 40)	*t-*value	*p-*value	*η^2^p*
1 h post-extubation	3.8 ± 0.5	2.1 ± 0.4	16.791	<0.001	0.783
4 h post-extubation	3.5 ± 0.4	1.7 ± 0.3	22.768	<0.001	0.869
12 h post-extubation	2.3 ± 0.4	1.1 ± 0.2	16.971	<0.001	0.787
24 h post-extubation	1.8 ± 0.3	0.7 ± 0.1	22.000	<0.001	0.861
48 h post-extubation	1.2 ± 0.2	0.5 ± 0.1	19.799	<0.001	0.834

η^2^p indicates partial eta-squared, representing the effect size; values of 0.01, 0.06, and 0.14 represent small, medium, and large effects, respectively.

### Adverse events

The incidence of adverse reactions between the two groups was compared and found to have no statistically significant difference. The types of adverse reactions monitored included nausea and vomiting, respiratory depression, pruritus, and local anesthetic toxicity. In the control group, the incidence of adverse reactions was as follows: nausea and vomiting in 3 patients (7.5%), respiratory depression in 2 patients (5%), pruritus in 1 patient (2.5%), and no cases of local anesthetic toxicity. In the combination group, the incidence of adverse reactions was nausea and vomiting in 2 patients (5%), respiratory depression in 1 patient (2.5%), pruritus in 1 patient (2.5%), and no cases of local anesthetic toxicity. The overall adverse reaction rate was 15% in the control group and 10% in the combination group. Statistical analysis revealed that these differences were insignificant (*p* > 0.05), indicating that adding esketamine to the QLB anesthesia regimen does not increase the risk of adverse events.

### Overview of statistical outcomes and effect sizes

Beyond mere statistical significance, the calculated η^2^p for all primary and key secondary outcomes—including perioperative MMSE scores, VAS pain scores, intraoperative anesthetic consumption, and recovery quality parameters—consistently exceeded the threshold of 0.14, indicating large effect sizes. Specifically, the substantial η^2^p values for MMSE scores on postoperative days 1 and 3 (0.173 and 0.238, respectively) and postoperative VAS scores (0.783–0.869) suggest that the combined intervention of esketamine and QLB exerts a robust clinical impact on cognitive preservation and analgesia. Furthermore, the large effect sizes observed in hemodynamic stability during critical periods (e.g., during pneumoperitoneum MAP η^2^p = 0.343) and recovery efficiency (e.g., time to spontaneous respiration η^2^p = 0.527) further underscore the high magnitude of the clinical benefit provided by this multimodal regimen. Importantly, while the efficacy was significantly enhanced, the incidence of adverse events (10% in the combination group vs. 15% in the control group) showed no statistical difference (*p* > 0.05), confirming that this potent synergistic strategy does not compromise the safety profile in frail older patients.

## Discussion

This study explored the effects of esketamine combined with QLB on postoperative anxiety, depression, and cognitive function in frail older patients undergoing radical colorectal cancer surgery. Our findings indicate significant benefits in hemodynamic stability, reduced anesthetic drug usage, improved recovery quality, cognitive function preservation, and superior pain management in the combination group.

Compared to the control group, the anesthetic approach in the treatment group effectively inhibits the connection between nociceptive stimuli and the sympathetic nervous system, thereby suppressing sympathetic excitation (24). This suppression reduces the secretion of catecholamines and blocks the transmission of nerve impulses at sympathetic nerve terminals, leading to decreased norepinephrine release (25). Consequently, this reduces nerve tension, lowers vascular pressure, and stabilizes the body’s hemodynamics (26). Therefore, while the baseline MAP and HR showed no significant differences between the treatment and control groups, the MAP and HR in the treatment group were significantly lower during post-induction, pneumoperitoneum, and postoperatively periods. These findings further confirm our initial hypothesis that the combined anesthetic technique provides superior hemodynamic stability by mitigating the physiological stress response to surgery.

The reduced dosages of remifentanil and propofol in the combination group highlight another significant advantage of the combined anesthetic approach. The combination group required significantly lower doses of these drugs, which can be attributed to the enhanced analgesic effects of QLB and the psychotropic properties of esketamine. This reduction in anesthetic requirements is crucial in minimizing potential side effects (27) and improving overall patient safety (28), particularly in older patients who are at higher risk for drug-related adverse effects (29).

Another advantage of combined esketamine anesthesia based on QLB is its ability to provide excellent postoperative analgesia with minimal impact on inflammatory factors in patients undergoing lower abdominal or pelvic surgery (19). This approach eliminates the need for opioids, which are commonly associated with various side effects such as respiratory depression, nausea, and constipation (19). By utilizing esketamine and QLB, patients experience effective pain relief without the adverse effects of opioids, while also maintaining a stable inflammatory response, which is crucial for optimal recovery and reduced postoperative complications (19).

Besides, patients in the combination group experienced significantly shorter extubation and spontaneous respiration times, along with lower agitation scores upon awakening, suggesting quicker recovery times and better postoperative outcomes with QLB and propofol anesthesia. The addition of esketamine, known for its rapid onset and short duration of action, further enhances recovery by reducing the time patients spend under the influence of anesthetics (30).

The improved recovery quality in our study is particularly important for older frail patients, as prolonged recovery times can lead to complications such as prolonged hospital stays, increased healthcare costs, and higher morbidity rates (31). The combination group offers a viable solution to mitigate these risks and enhance postoperative recovery.

The preservation of cognitive function, as evidenced by higher MMSE scores in the combination group on postoperative days 1 and 3, is a significant finding. Cognitive dysfunction is a common and debilitating complication in older patients after major surgery (32). The use of esketamine, with its neuroprotective and antidepressant properties, combined with the regional analgesic effects of QLB, appears to mitigate this risk (33).

This result is consistent with previous research that highlights the benefits of regional anesthesia in preserving cognitive function (32). Studies have shown that regional blocks, such as QLB, reduce the incidence of postoperative cognitive dysfunction (POCD) by minimizing systemic opioid use and providing targeted analgesia (33). The addition of esketamine further supports cognitive health by modulating neural pathways involved in mood and cognition (33).

VAS scores for pain were significantly lower in the combination group at all postoperative time points (1, 4, 12, 24, and 48 h) compared to the control group. Effective pain management is crucial for postoperative recovery, especially in older patients who may have diminished pain tolerance and increased sensitivity to analgesic side effects (33, 34). The superior pain control observed with the combination group is consistent with findings from other studies (19). These findings collectively support the use of QLB in conjunction with other analgesics to enhance postoperative pain management.

The superior perioperative outcomes observed in the combination group may be attributed to the multifaceted synergistic mechanisms between esketamine and the posterior QLB. From a neurobiological perspective, this multimodal strategy targets pain processing through a dual-pathway modulation model. QLB provides potent somatic and visceral analgesia by intercepting nociceptive transmission at the spinal nerve roots and the sympathetic trunk, effectively attenuating the ‘bottom-up’ afferent signaling (15). Simultaneously, esketamine, the S(+)-enantiomer of ketamine, acts as a high-affinity, non-competitive antagonist of the N-methyl-D-aspartate (NMDA) receptor, binding specifically to its phencyclidine site to block calcium influx (35). This exerts a ‘top-down’ regulatory effect that inhibits excitatory glutamatergic transmission, thereby mitigating central sensitization and the ‘wind-up’ phenomenon, which is often exacerbated in older frail patients with diminished neural plasticity.

The clinical utility of this regimen is further underscored by the unique pharmacodynamic profile of esketamine. Unlike traditional sedative-hypnotics, esketamine induces ‘dissociative anesthesia’ while maintaining sympathetic tone and upper airway patency (36). This characteristic explains the enhanced hemodynamic stability observed during high-stress periods, such as pneumoperitoneum. Notably, we addressed the potential pharmacological impact of esketamine on muscle tone. While central excitatory-inhibitory imbalance can slightly increase skeletal muscle tone, our findings suggest this did not impede surgical conditions. On the contrary, by facilitating a robust ‘top-down’ analgesic effect, the combination group required significantly lower doses of opioids and propofol. This reduction in the cumulative ‘sedative burden’, a known risk factor for postoperative cognitive decline (POCD), likely counteracted any myogenic effects, resulting in accelerated recovery of spontaneous respiration and shorter extubation times (37).

The efficacy of NMDA antagonism is particularly relevant given the age-related alterations in receptor density and sensitivity (38). Although older adults exhibit a global decrease in NMDA receptor density in the prefrontal cortex and hippocampus, the remaining receptors in the pain-processing pathways often demonstrate heightened sensitivity to perioperative neuro-inflammatory stimuli. By stabilizing these receptors, esketamine prevents calcium-mediated neuronal apoptosis and suppresses the release of pro-inflammatory cytokines, thereby preserving blood–brain barrier integrity (39). This ‘neuro-buffering’ effect is analogous to recent findings in obstetric research, where low-dose esketamine has been shown to prevent postpartum depression by modulating the NMDA-glutamate system during the acute stress of childbirth. We speculate that older frail patients undergoing colorectal surgery face a similar ‘neuro-inflammatory hit’; for these individuals, the rapid-acting antidepressant-like properties of esketamine, mediated by the AMPA-mTOR signaling pathway and increased brain-derived neurotrophic factor (BDNF) expression, may enhance neuro-resilience, effectively mitigating the psychological stress and agitation that often precede cognitive impairment.

Finally, our results suggest that the benefits of the esketamine-QLB regimen are particularly nuanced by patient-specific frailty levels and surgical characteristics. Subgroups with higher frailty severity, characterized by lower physiological reserves, may derive the most profound neuroprotective benefits from this intervention (40). Furthermore, for patients undergoing prolonged laparoscopic procedures involving intense peritoneal stretching and metabolic stress, this multimodal approach is advantageous for mitigating massive sympathetic activation. By integrating potent regional blockade with central NMDA modulation, this strategy provides a scientific foundation for individualized perioperative pathways, effectively transforming pharmacological synergy into improved neuropsychological outcomes in the vulnerable older surgical population.

However, our study also has some limitations that should be considered when interpreting the results. First, while the sample size was adequate for detecting significant differences in our primary outcomes, such as the MMSE scores and intraoperative anesthetic consumption, it remains relatively small. Larger, multicenter trials are required to confirm these findings and enhance their generalizability. Second, as a single-center study, the clinical protocols and patient demographics may limit the applicability of our results to other institutional settings.

Third, although the outcome assessors and patients were strictly blinded, the anesthesiologist performing the QLB and administering esketamine was not blinded due to the nature of the clinical intervention, which may introduce a potential performance bias. Fourth, our study focused on the immediate perioperative period and short-term recovery (up to postoperative day 3). Therefore, the long-term effects of this multimodal regimen on cognitive trajectories at 3 or 6 months remain unknown. Finally, while we observed significant benefits in our cohort, further research is needed to determine whether different levels of frailty severity or specific surgical approaches (e.g., robotic vs. laparoscopic) influence the magnitude of the synergistic effect between esketamine and QLB.

Future research should focus on optimizing the dosing regimen of esketamine and exploring its long-term effects on cognitive function and psychological well-being. Investigating the combined use of QLB with other non-opioid analgesics could further enhance postoperative recovery and reduce the reliance on systemic opioids. Additionally, studies should explore the potential benefits of QLB and esketamine in other high-risk surgical populations, such as those with multiple comorbidities or advanced malignancies.

## Conclusion

This study demonstrates that esketamine combined with QLB significantly improves postoperative outcomes in frail older patients undergoing radical colorectal cancer surgery. This combined approach enhances hemodynamic stability, reduces anesthetic drug requirements, improves recovery quality, preserves cognitive function, and provides superior pain management. These findings support the clinical adoption of this anesthetic strategy to optimize perioperative care in this vulnerable patient population. Further research is warranted to confirm these benefits and explore additional applications of this approach in other high-risk surgical groups.

## Data Availability

The original contributions presented in the study are included in the article/supplementary material, further inquiries can be directed to the corresponding author.
